# The Institutional Worker

**Published:** 1918-09-21

**Authors:** 


THE
### INSTITUTIONAL WORKER
**Being a Special Supplement to "The Hospital"**

### OUR BUREAU OF INFORMATION.

### Rules for Correspondents.

1. Every letter must be accompanied by the coupon to be cut from the back cover (inside page) of The Hospital, current issue, and must contain the name and address of the correspondent with pseudonym for publication if desired. Replies by post cannot be given save under exceptional circumstances at the Editor's discretion.
2. Letters from Approved Homes in reply to special needs published in the Bureau should state terms and full particulars, and be sent prepaid under cover to the Editor of the Bureau with name written across coupon for identification.
3. Proprietors of Homes which have not yet been entered on the List of Approved Homes, but have spare accommodation likely to suit special needs, are invited to write for an application form for registration. The fee for registration, which includes two announcements of the Home in the Bureau and other privileges, is 10s.
4. All communications to be addressed to the Editor of The Hospital, 28 Southampton Street, Strand, London, W.C. 2, and marked "Bureau of Information."

### INSTITUTION FACTS AND FIGURES.

### A Burning Question.

#### PEAT AS A COAL SAVER: ITS CULTIVATION AND USE.

Shortage of coal has raised the question of utilisation of our resources in peat, and a Board of Trade circular dated July 1 urges local effort and the utilisation of such local knowledge of peat-winning as still remains, adding a few simple instructions. In Sussex, when Lancing College was built, two old men were discovered in the county who had some knowledge of the beautiful craft of facing buildings with flint, and a noble revival was the result. It will be well if something can be done before next winter, when to be able to say, "Aha, I am warm, I have seen the fire!" may not happen to us daily. English bogs have never been fully surveyed. The 30,000,000 acres known to exist in Canada and the 20,000,000 in the U.S.A. will in their turn prove useful. Ireland's store of something less than 3,000,000 acres has, of course, been worked for generations. The question of quality will arise. The black bog of a low-lying Irish valley, "bottom peat," is better and less friable than the lighter brown "hill peat," or, rather, "turf;" as it is more often called there. "A few turf" is the accepted plural. Reeds, rushes, sedges, mosses usually combine in water that is still but not quite stagnant to form bog, in a mean temperature of about 45° F. It is remarkable that none is known to exist outside a zone of 45° N. and 45° S. latitude. Occasionally in Ireland it has happened that a half-set bog has overflowed the depression in which it was formed and "travelled" dangerously. Most valley bogs contain pockets of wet stuff—"shaky scraws"—clothed with deep moss or vivid green grass, which warn natives of the danger of stepping on them.

Wood for Kindling Unnecessary.

When cut and set to dry the sods may contain much water, even up to 90 per cent.; consequently, the drying should be finished before frost can disintegrate the substance. Turf fires have the great advantage of dispensing with wood for kindling. Leave two sods to keep each other warm, or even one, face down on the warm hearth, and your bellows in the morning will renew the fire. But a little skill must be learnt. A writer in the *Times* spoke lately of a fine blaze produced by "throwing on" peat. The expert never throws it on; each sod is placed —it will not coalishly blacken the hands if touched—to allow air to pass. Its pleasant odour and blue smoke are famous. Another merit is that the proportion of sulphur is much less than in coal. But it cannot rival good coal in giving out heat, and M. Ekenberg devised a plan of pulping, milling, carbonising, and compressing it into solid briquettes—unfortunately, a costly process. It has also been reduced to good coke, with ammonium sulphate as a by-product, and experiments by Sir William Ramsay and R. W. Wallace showed that 25 to 26 gallons of motor spirit could be extracted from one ton of peat. In 1906 a factory was established at Capac, Michigan, to produce wrapping-paper of good quality from peat by C. E. Nelson's process. Experiments in weaving the delicate tufts of "bog cotton," however, have not succeeded, owing to the shortness of the staple.

In Kitchen and Sitting-room.

But our immediate concern is with peat winning and burning. The first is not hard, and could be done by women. The half-spade, "slane" or "loy" which is worked in Ireland by either right or left foot, according to its shape, cuts the sod like cake. Spread singly and then piled three or so together, with air passing between, these sods must be turned once or twice to dry every part, and then stacked. The question of the grate is, however, important. The flat hearth or Nautilus grate, or the old-fashioned, over-roomy Victorian one, which we perforce diminish with fire-brick, will serve the purpose, but hardly the narrow type of "parson's" grate. Peat takes room and needs draught, or it will smoulder dismally. Wood used with it makes a gay fire. Its white ash makes what the Irish housemaid wisely calls "clane dirt" as compared with the grime of coal. It will not give out spurts of gas as coal does, although gas has been extracted from it both in England and in Germany. For cooking it worked well in the old-fashioned open range and under hot plates for *petit feu*, and has, of course, the advantage that a little fire can be kept going till more is needed. But experiment will be needed for its use in our modern coal-saving cookers.

Meanwhile there is no time to be lost for the drying process before frosts and autumn rains interfere with peat-winning for next winter. Many English bogs, too, are much overgrown, and clearing will be needed. It is a new field for those who are taking "working holidays."

### The Salvage of Refuse.

Some of the more enterprising municipal authorities have made provision for the collection of refuse of all sorts. Much, however, remains to be done in this direction, and the funds of hospitals might be benefited to a greater extent than they are by the salvage and sale of waste. The difficulty is that the individual hospital cannot produce sufficient of some of these materials to be regarded as more than negligible. As we have previously suggested, an extraneous body—such as, for instance, the Hospital Saturday Fund—might arrange for a depôt or depôts for the accumulation and sale of waste, not only from the hospitals, but from private houses. This should not be a difficult matter as regards the hospitals in the Metropolitan area. Among the many items could be included old electric lamps, rags, bones, tins, soiled dressings—which are now being cleaned, sterilised, and used for suitable purposes—old instruments, waste paper, and old magazines and newspapers, which accumulate enormously in every hospital.—Moir.

### SEPTEMBER QUESTION.

### Fuel and Lighting Economies.

**1. Describe means by which economies may be effected in the consumption of fuel and lighting in an institution (a) in the wards, ward kitchens, etc., (b) in the kitchens and servants' quarters, (c) in the Nurses' Home, (d) in the administration building.**

**2. What steps may be taken in the engineer's department to keep up the heating and lighting and hot-water supplies with the least possible expenditure of fuel?**

**(A minimum payment of 10s. 6d. will be made for each published answer. It is not imperative for both parts of the question to be answered.)**

### RULES.

**The following rules must be observed:—**

**1. Contributions must be written on one side of the paper. Brevity and terseness are desirable features. The MSS. must bear the name and address of the sender and be accompanied by coupon to be cut from the back cover (inside page) of the current issue of THE HOSPITAL. A pseudonym must be chosen if the name is not to be published.**

**2. Contributions must be addressed to the Editor of THE HOSPITAL, Institutional Worker Supplement, 28 & 29 Southampton Street, Strand, London, W.C. 2, should reach him before the end of the current month, and be marked in the left-hand corner "Facts and Figures."**

### QUESTIONS INVITED.

**IN connection with our Question Box, we offer every month five shillings each for the two best questions which are sent in for consideration. The questions may be on any institutional subject and concern any department, from the secretary's to the porter's, or the matron's to the domestic staff; the one essential is that they must be practical and deal with points that have a definite relation to hospital or institutional administration, upkeep, finance, or management. Points relating to artificers' work, housekeeping, the laundry, out-patients, and other practical matters will be welcome. It is hoped that thus, when difficult or doubtful points arise in the course of their work, institutional workers will be encouraged to put them in the form of a question and send them to the Editor, THE HOSPITAL Bureau of Information, 28 & 29 Southampton Street, Strand, W.C. 2, marked "Question Box."**

**The best questions will be published in due course and our readers will be given the opportunity of answering them. Thus all institutional workers may be able to cooperate with the view of helping the work and smoothing the difficulties of each other. In short, we want the Question Box of the *Institutional Worker* to be freely used by every worker habitually.**

### ENQUIRIES AND ANSWERS.

### *THE SICK AND IN NEED.*

### Home for Feeble-Minded Child.

As you do not give many details of the case in question it is somewhat difficult to suggest a suitable home. However, we give you the addresses of a few you might write to :—Home for Feeble-Minded Girls, Handford House, Ranelagh Road, Ipswich; feeble-minded girls of good character and capable of improvement are taken and trained in house and laundry work. Payment of 10s. a week is required. Apply to the Hon. Secretary. The Horticultural School for Feeble-Minded Girls, Dovecot, Knotty Ash, Liverpool; a certified home for permanent care of feeble-minded girls, who are trained in horticulture, house and laundry work. Apply to the Hon. Secretary; payment of 10s. to 14s. weekly and £3 for outfit is required. The Laundry and Home of Industry, Bow Villa, Morpeth; girls up to ten years of age are accepted at a charge of 8s. per week. You might also apply to the Mary Carpenter Home, Causeway, Fishponds, Bristol. The object of the home is to protect and train feeble-minded girls between the ages of fourteen and twenty-four. Payment, 10s. weekly.—S. M. W.

### Convalescent Home for Woman.

POSSIBLY one of the following homes will be suitable for the poor woman you wish to send to a convalescent home :—The London and Brighton Female Convalescent Home, Crescent House, Marine Parade, Brighton. Admission is by subscriber's letter and payment of 9s. per week; if a compartment is shared with another the charge is 11s. 6d.; if a private compartment is required 13s. 6d. per week. Cases of mental derangement, fits, or infection are ineligible. The Victoria Wellesley Home, Walton Road, Bognor. Admission is by letter and 5s. weekly. Application must be made to the Lady Superintendent. The Rest and Convalescent Home for Women, Rest Lodge, Bognor, receives women needing only rest and bracing air by payment of 10s. 6d. to 18s. weekly.—CLERGY.

### Home for Inebriate Woman.

YOU might apply to the Secretary of the House of Refuge, Ingleston, Greenock, if you have not already done so, on behalf of the woman. The home is licensed for thirty inebriates; admission is free.—RESCUE WORKER.

### Home for Soldier's Orphan.

A HOME might be found for the child who has recently lost both parents at the Royal Victoria Patriotic Asylum, Wandsworth Common, S.W. Children are admitted between the ages of seven and eleven, and are given a sound elementary and religious education and practical training for domestic service. During the occupation by the military of the asylum, the inmates reside in rented houses at Spencer Park, Wandsworth Common.—ALSTON.

### *EMPLOYMENT AND TRAINING.*

### Training of Pharmacists.

FACILITIES are provided at the Battersea Polytechnic for the complete educational training of pharmacists. Those who have not passed a Preliminary Arts Examination recognised by the Pharmaceutical Society can join day or evening classes for one or all of the requisite subjects. Students must serve an apprenticeship with a chemist and druggist or a pharmaceutical chemist for a period of three years, and on attaining the age of twenty-one can enter for the Minor Examination of the Pharmaceutical Society. The holder of the Minor certificate is entitled to style himself chemist and druggist. The subjects of this examination include chemistry, pharmacy and prescription-reading, materia medica, and botany. The certificate of the Major Examination confers the title of pharmaceutical chemist. The scheme of education provided by the Battersea Polytechnic enables the student either to commence a three years' course of study in the evening directly he has passed the Preliminary Examination or to attend a sessional course and devote his whole time to study for a session of nine months. The fee for the sessional course is £13 13s., September to June. Full particulars may be obtained upon application to the Principal, Battersea Polytechnic, Battersea Park Road, S.W. 11.—B. C.

### *THE HOUSEKEEPERS' DEPARTMENT.*

### Old Rags.

THE demand for old rags for paper-making is greater now than ever, and some local authorities are giving this matter their special attention. Reliable buyers offer good prices. Bones are especially valuable now, and should on no account be consigned to the refuse destructor.—B. W.

### *MISCELLANEOUS.*

### How to Enlighten our Children.

THE address by Mr. Stephen Paget given at the Oxford University Extension Meeting in August 1917 on this subject is published by Constable (price 7d.).—GWENT.

### Repairs of X-Ray Tubes.

IT is not easy at the present time to get *x*-ray tubes repaired expeditiously. Among the several makers in London who carry out this work are H. Helm, 66 Hatton Garden, E.C. 1. We do not advise the repair of tubes which have been used extensively, as the residual gases in the metal parts are exhausted by long use, and consequently the life of the tube when repaired would be short. The makers will advise you upon this point, however.—T. T.

